# Comparison of the serological tests ICT and ELISA for the diagnosis of alveolar echinococcosis in France☆

**DOI:** 10.1051/parasite/2014037

**Published:** 2014-07-25

**Authors:** Jenny Knapp, Yasuhito Sako, Frédéric Grenouillet, Solange Bresson-Hadni, Carine Richou, Houssein Gbaguidi-Haore, Akira Ito, Laurence Millon

**Affiliations:** 1 Laboratory of Chrono-environnement, UMR/CNRS 6249, Faculty of Medicine and Pharmacy Besançon France; 2 WHO Collaborating Centre for prevention and treatment of human echinococcosis Besançon France; 3 Department of Parasitology, Asahikawa Medical University Asahikawa Hokkaido Japan; 4 Laboratory of Parasitology-Mycology, University Hospital of Besançon France; 5 Department of Hepatology, University Hospital of Besançon France; 6 Laboratory of Hospital Hygiene, University Hospital of Besançon France

**Keywords:** alveolar echinococcosis, diagnosis, rEm18, immunochromatography, rapid test, rEm18-ELISA and Em2-Em18-ELISA tests

## Abstract

Serological diagnosis of alveolar echinococcosis (AE) is a key element for efficient patient treatment management. A rapid immunochromatography test kit (ICT) using the recombinant Em18 antigen (rEm18) was recently developed. The aim of our study was to assess this test on a panel of sera from French patients with alveolar echinococcosis and control patients. In a blind test, a total of 112 serum samples were tested including samples of AE (*n* = 30), cystic echinococcosis [CE] (*n* = 15), and polycystic echinococcosis [PE] (*n* = 1). For the comparison, 66 sera from patients with hepatocarcinoma, fascioliasis, toxocariasis, Caroli’s disease, or autoimmune chronic active hepatitis were used. The diagnostic test sets we used were the rEm18-ICT and two validated ELISAs with rEm18 and Em2-Em18 antigens, respectively. For the ICT, 27/30 sera from AE patients, 4/15 sera from CE patients and the PE patient serum were positive. One serum from the control panel (toxocariasis) was positive for the ICT. The rEm18-ICT sensitivity (90.0%) and specificity (92.7%) for detection of Em18-specific antibodies confirmed it as a relevant tool for AE diagnosis. The rEm18-ELISA had a sensitivity of 86.7% and specificity of 91.5%, and the Em2-Em18-ELISA had a sensitivity of 96.7% and specificity of 87.8%. However, when AE patient sera are recorded as weak in intensity with the ICT, we recommend a double reading and use of a reference sample if the ICT is used for patient follow-up.

## Abstract (fr)

Le diagnostic sérologique de l’échinococcose alvéolaire (EA) est un point fondamental pour assurer l’organisation du traitement du patient. Un test rapide immuno-chromatographique (ICT), utilisant l’antigène recombinant Em18 a été récemment développé. Le but de cette étude était d’évaluer ce test sur un panel de sérums de patients français présentant une échinococcose alvéolaire et sur des patients de contrôle. Lors d’un test réalisé en aveugle, un total de 112 sérums a été testé, regroupant des échantillons d’EA (*n* = 30), d’échinococcoses kystiques (EK) (n = 15) et d'échinococcose (EP) (*n* = 1). Pour comparaison, 66 patients présentant un hépatocarcinome, une distomatose, une toxocarose, une maladie de Caroli ou une hépatite active chronique auto-immune ont été utilisés. Les tests diagnostiques employés étaient l’ICT rEm18 et deux tests ELISA validés utilisant respectivement les antigènes rEm18 et Em2-Em18. Avec l’ICT, 27/30 sérums de patients EA, 4/15 EK et le patient EP étaient positifs. Un sérum du panel contrôle (toxocarose) présentait un ICT positif. Pour le test ICT rEm18, la sensibilité (90,0 %) et la spécificité (92,7 %) pour la détection de l’antigène Em18 confirme que ce test est un outil fiable pour le diagnostic d’EA. L’ELISA rEm18 présentait une sensibilité de 86,7 % et une spécificité de 91,5 %, et l’ELISA Em2-Em18 une sensibilité de 96,7 % et une spécificité de 87,8 %. Néanmoins lorsque des sérums de patients présentent une bande test d’intensité faible pour l’ICT nous recommandons une double lecture et l’emploi d’un échantillon de référence pour une utilisation en suivi.

## Introduction

In rodents and humans, alveolar echinococcosis (AE) appears as a tumor-like lesion and is caused by accidental ingestion of eggs of the cestode *Echinococcus multilocularis*, a fox tapeworm. The adult worm produces eggs which are released into the environment with fox feces. The parasite is widely present in the Northern Hemisphere in countries such as China, where the estimated prevalence ranges from 0.2% to 9% in 12 regions [22]. The parasite is also present in temperate Europe, where a total of 559 human cases have been identified in nine countries [9, 14]. A recent increase in reported cases of human AE and of *E. multilocularis* in wild animals has been observed not only in historically endemic regions in Europe [6, 20], but also in new endemic regions [5, 23]. In France, from 1982 to 2009, 8 to 29 new cases per year, mostly in the Northeast, were identified by the FrancEchino network [12]. The exposure to eggs is likely due to repeated contact with wild or domestic carnivores such as foxes, dogs, and cats [21], consumption of wild berries or raw vegetables growing close to the ground, and agricultural activities [3]. The main AE symptoms are abdominal pain, asthenia, and hepatomegaly. Generally, the first symptoms appear 5–15 years after contamination [1, 3]. Diagnosis is often made based on images obtained by ultrasound, computerized tomography, or magnetic resonance imaging [4]. Immunodiagnosis tests, e.g., the enzyme-linked immunosorbent assay (ELISA) using rEm18 (rEm18-ELISA) [18] or rEm18 plus the native Em2 antigen purified from *E. multilocularis* larvae (Em2-Em18-ELISA) (Bordier Affinity, Crissier, Switzerland), are currently being used in laboratories. Indirect hemagglutination (IHA) (Hydatidose Fumouze kit, Fumouze Diagnostics, Levallois-Perret, France) is one of the low-cost screening techniques [11], and the Western blot technique (WB) (LDBIO Diagnostics, Lyon, France), using a whole *E. multilocularis* larval antigen, is the confirmation technique for species diagnosis [4, 16].

In 2003, Xiao et al. demonstrated the specificity of rEm18 for AE diagnosis using serum samples from patients with other parasitic infections and hepatic diseases [24]. In addition, they demonstrated that measurement of rEm18-specific antibodies can give information on parasite status after implementation of treatment [13], because antibody response against this recombinant antigen reflects the activity of the parasite. Recently, an immunochromatography test (ICT) using the rEm18 antigen was developed [17] and a sensitivity of 94% and a specificity of 95.4% were found for AE sera. This kit is commercially available now (ADAMU-AE kit, ICST Co. Ltd., Saitama, Japan).

The main aims of our study were to assess the reliability of the ICT results in the detection of AE cases using a panel of French sera, by comparing the ICT with ELISA tests, which are validated and routinely used in laboratories, and to assess rEm18-ICT reproducibility on different batches of kits.

## Materials and methods

### Serum samples

A total of 112 serum samples were collected from patients. Sera were received from 1987 to 2010 at the Parasitology Department (University Hospital, Besançon, France) for diagnosis of *Echinococcus* and other pathologies. The *Echinococcus* panel (46 samples) was composed of 30 AE (29 with liver lesions as a primary focus, and one with a lung lesion), 15 CE, and 1 polycystic echinococcosis (PE) (due to *E. vogeli* infection) [15]. Only AE cases based on the consensual criteria established by Brunetti et al. were included. Diagnosis had been carried out by clinical findings, imaging techniques, serology with Western Blot (LDBIO, Diagnostics, Lyon, France), specific PCR and/or histology [4]. The sera of AE patients were sampled before any parasitostatic treatment. The control serum collection (66 samples) was composed of 13 toxocariasis, 13 hepatocellular carcinoma, 8 fascioliasis, 7 autoimmune systemic diseases with high levels of circulating rheumatoid factors, 7 Caroli’s disease, 5 autoimmune chronic active hepatitis, and 13 other pathologies involving the liver, i.e., liver cysts (*n* = 3), biliary cirrhosis (*n* = 3), angioma (*n* = 2), metastasis of breast carcinoma (*n* = 1), cystadenocarcinoma (*n* = 1), bacterial abscess (*n* = 1), bile duct carcinoma (*n* = 1), and neurocysticercosis (*n* = 1). For the fascioliasis and toxocariasis cases, the serology (specific serum antibodies assessed by highly sensitive serological tests, and confirmed by WB, a separate high specificity serological test) was classified as positive by clinical and epidemiological history (LDBIO Diagnosis, Lyon, France).

### rEm18 Immunochromatography Test

The rEm18-ICT was based on the detection of antibodies against the recombinant Em18 antigen (rEm18) [18]. The antigen and anti-goat immunoglobulin G (IgG) as test and control lines, respectively, were sprayed onto a nitrocellulose membrane, and placed in a plastic device [17]. For the assay, first, 10 μL of the serum sample were mixed in a tube with 20 μL of a serum dilution buffer containing 0.1 mg/mL alkaline phosphatase-conjugated goat anti-human IgG antibody (DAKO, Tokyo, Japan). This mixed serum sample was then applied onto the sample window of the plastic device, and within 30 s, 200 μL of the substrate solution was loaded and the result was determined after 30 min. For color development, 5-bromo-4-chloro-3-indolyl-phosphate was used. A sample was considered positive if two color lines were present after 30 min, indicating the presence of the specific anti-recEm18 antibodies (test line: rEm18), and if the control line (anti-goat IgG) was visible (indicating that the test was performed correctly). Because the ICT is a manual test, the cut-off has to be determined by visual observation. In order to obtain objective ICT results for this study, two different persons independently inspected the bands that appeared on the ICT device. After that, for the positive tests, an index intensity was calculated to enable comparison of the test results. For all samples, a picture was taken using a ChemiDoc apparatus (Bio-Rad laboratories, Hercules, CA, USA) and the intensities of the control and test bands and of the background membrane were recorded, after exposure to Epi-white light (Quantity One 4.6.5 software). The relative intensity index was calculated so that batches used for the same sera could be compared. The index intensity calculation in our study differs from that of Sako et al. [19] because there is no immunochromato-reader in the Chrono-Environnement Laboratory at the Faculty of Medicine and Pharmacy, Besançon, France.

The background membrane intensity was subtracted from the test and control band intensities, yielding the following formula:

Index intensity = (test band intensity - background intensity) / (control band intensity - background intensity).

### Em2-Em18-ELISA and rEm18-ELISA

The Em2-Em18-ELISA detects IgG antibodies in human serum against Em2 and Em18 antigens (Bordier Affinity, Crissier, Switzerland). IgG antibodies were detected with a protein A-alkaline phosphatase conjugate. Microtitration was assessed by ELISA on an Evolis microplate automaton (Bio-Rad Laboratories, Hercules, CA, USA). The cut-off value calculation was established as recommended by the manufacturer, and the “home index” was considered as a positive value when 60% of the recommended index value was attained. Thus, a high sensitivity level was achieved, while maintaining good specificity.

The rEm18-ELISA was performed as previously described [18]. Optical density (OD) was measured and the cut-off value was 0.12. This cut-off value was calculated from the OD values from 40 negative controls (healthy people with no clinical features of AE). The average was calculated and 4 standard deviations were added to provide the cut-off value. Serum samples were sent to Japan and analyzed in a blind test at the Department of Parasitology, Asahikawa Medical University, Hokkaido, Japan.

### Statistical analysis

Overall reproducibility was tested with the Friedman test, and pairwise comparisons were tested with the Wilcoxon signed-rank test. Indicators of diagnostic test performance were: sensitivity (Se), specificity (Sp), the Youden index (YI = Se + Sp − 1), to assess the efficiency of the test (negative value for an inefficient test, positive value for a reliable test), and accuracy (proportion of patients correctly classified). Positive and negative likelihood ratios (LR+ and LR−), which respectively describe the discriminatory abilities of positive and negative test results, were calculated, with a 95% confidence interval (95% CI). A LR+ value above 10 and a LR− value below 0.1 were used to confirm the relevance of the diagnostic tool [8]. The diagnostic odds ratio (DOR = LR+/LR−) was also determined. The DOR can be used on its own to indicate a test’s discriminatory performance [10]. All analyses were two-tailed, and a *p*-value of less than 0.05 was considered significant. The software package Stata 10.0 (StataCorp LP, College Station, TX, USA) was used for the analysis.

## Results

### rEm18-ICT: sensitivity, specificity and reproducibility

**Figure 1.**
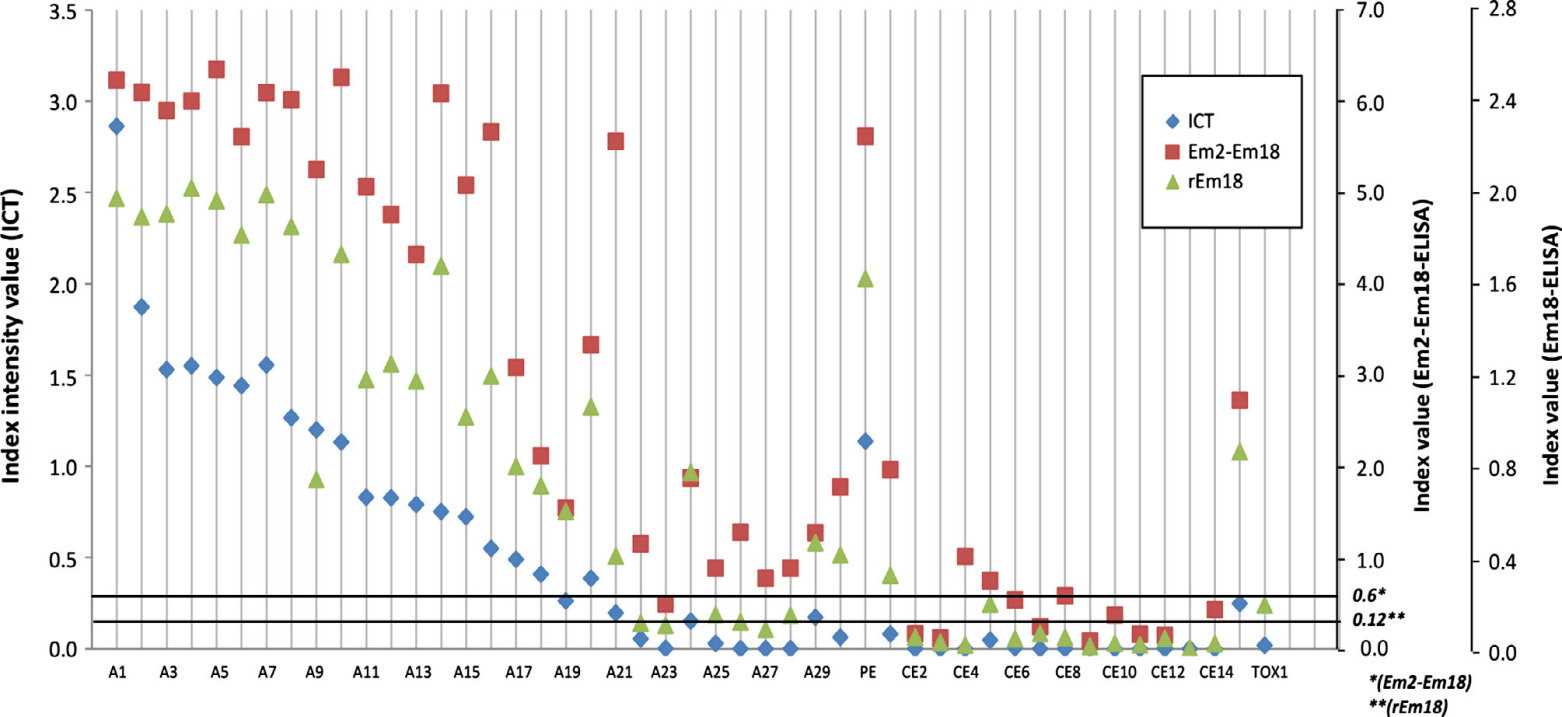
Immunological response to *E. multilocularis* antigens. Comparison of rEm18-ICT with ELISA rEm18 (OD values) and Em2-Em18 (index values) for AE (alveolar echinococcosis), CE (cystic echinococcosis), PE (polycystic echinococcosis), and toxocariasis (TOX1) cases. The thin dotted line represents the Em2-Em18 index threshold and the thick dotted line represents the rEm18 OD threshold; *Em2-Em18 threshold index, **rEm18 threshold OD value.

Twenty-seven out of 30 AE sera were positive with rEm18-ICT as observed visually by independent double reading, demonstrating a sensitivity of 90.0% (Table 1 and Fig. 1). Four of the 15 CE cases tested were positive for the ICT; the PE case was also positive. One of the 66 control sera was positive (1 toxocariasis, in Fig. 1: TOX1).

**Table 1.** Results for ELISA tests rEm18 and Em2-Em18, and rEm18-ICT for AE (alveolar echinococcosis), CE and PE (cystic and polycystic echinococcosis), and other pathologies. Percentages of sensitivity, specificity, and the performance index for each test are shown.

		No. of seropositive samples for the three tests		
	No. of samples	rEm18-ICT	rEm18-ELISA	Em2-Em18-ELISA
AE	30	27	26	29
CE/PE	16	5	4	5
Other	66	1	3	5
Sensitivity, %		90.0	86.7	96.7
(95% CI)		(78.4–96.1)	(74.4–93.9)	(85.8–99.4)
Specificity, %		92.7	91.5	87.8
(95% CI)		(88.4–94.9)	(87.0–94.1)	(83.8–88.8)
Youden index		0.83	0.78	0.85
(95% CI)		(0.67–0.91)	(0.61–0.88)	(0.70–0.88)
Accuracy, %		92.0	90.2	90.2
(95% CI)		(85.7–95.2)	(83.6–94.1)	(84.4–91.6)
Positive likelihood ratio (LR+)		12.3	10.2	7.9
(95% CI)		(6.7–18.9)	(5.7–16.0)	(5.3–8.9)
Negative likelihood ratio (LR−)		0.11	0.15	0.04
(95% CI)		(0.04–0.25)	(0.06–0.30)	(0.01–0.17)
Diagnostic odds ratio		114.0	69.6	208.8
(95% CI)		(27.7–461.2)	(19.4–248.5)	(31.5–1314.4)

Thirty-three sera out of the 112 tested were ranked positive by rEm18-ICT with 27 specific diagnoses, with a specificity of 92.7 (Table 1).

**Figure 2.**
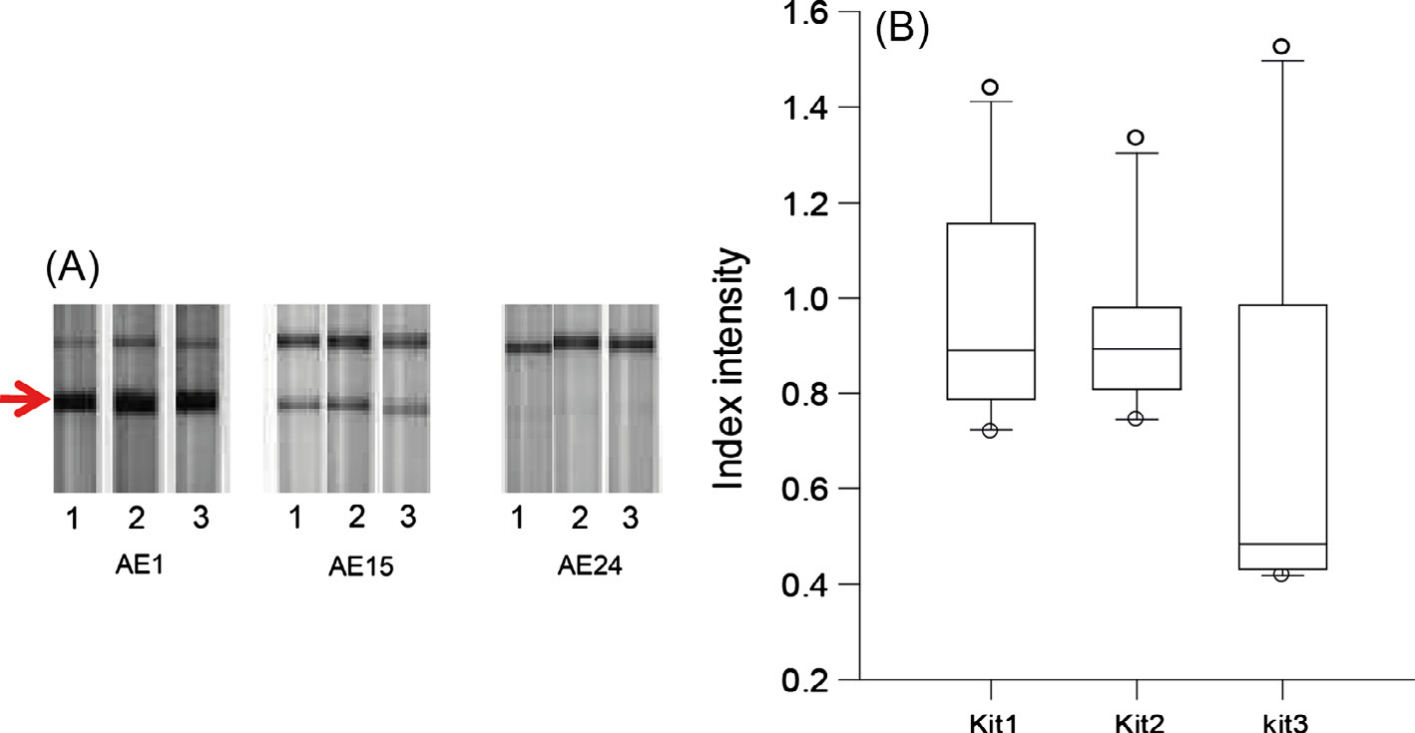
Test of reproducibility of rEm18-ICT using sera from 10 French AE (alveolar echinococcosis) patients, with three batches of kits (numbers 1, 2, and 3). (A) Strong, middle, and weak signals are shown for patients AE1, AE15, and AE24. The arrow shows the test band that indicates a positive test. (B) Index intensity values of three batches of kits; *p* < 0.05.

The reproducibility of band intensities was assessed on 10 different sera tested with three different batches of kits (Figs. 2A–2B). A significant difference was observed between batch 3 and the two others (*p* = 0.025). A significant difference was observed between batches No. 3 and No. 1 (*p* = 0.0093) and between No. 3 and No. 2 (*p* = 0.0125).

### ICT versus ELISAs

Thirty-three cases, including 26 AE, 3 CE, 1 PE, and 3 other diseases (liver cyst, toxocariasis [TOX1], and hepatocellular carcinoma), similar to the rEm18-ICT results (Table 1 and Fig. 1), were ranked seropositive by rEm18-ELISA, and 39 cases, including 29 AE, 4 CE, 1 PE, and 5 other diagnoses (liver metastasis, breast carcinoma, hepatocellular carcinoma, two cases of fascioliasis, and autoimmune chronic active hepatitis) were ranked seropositive by Em2-Em18-ELISA. There were no differences in diagnostic performance among the tests evaluated.

No significant differences were found in sensitivity and specificity among the methods used. (Fig. 1 and Table 1).

Five results were discordant between rEm18-ICT and the ELISAs for the diagnosis of AE. For one case, the ICT was weakly positive, whereas the two ELISAs were negative (AE23 in Fig. 1). For two cases, rEm18-ICT and rEm18-ELISA gave a negative result, and Em2-Em18-ELISA was positive (AE26 and AE27). For one case, rEm18-ELISA was negative, and rEm18-ICT and Em2-Em18-ELISA were positive (AE22). For the fifth case, the ICT was negative and the ELISAs were positive (AE28). The toxocariasis serum TOX1, positive with rEm18-ICT, was also positive with rEm18-ELISA, but not with Em2-Em18-ELISA.

For the ICT, the values of the LRs (LR+ = 12.3 and LR− = 0.11) confirmed the relevance of this diagnostic tool. In addition, the DOR, Youden index, and accuracy emphasized the relevance of the ICT for AE diagnosis and the reliability of its results compared with the other tests (Table 1), because no significant difference was observed.

## Discussion

Previous evaluations have revealed that the rEm18-ICT is a reliable and rapid test, applicable for AE diagnosis at first screening. It is worth noting that in the field, it is particularly handy because the kit comes in a plastic case. Our study focused on the usefulness of the ICT for routine serological diagnosis. The ICT provided good scores for specificity, sensitivity, the Youden index, accuracy and the LRs. In addition, DOR analysis confirmed that the ICT performs similarly to validated tests, i.e., ELISA. However, our results suggest that a combination of different serological tests seems to be necessary for an accurate diagnosis, especially for low immunological responses (Fig. 2A). The sera were tested in order to assess the usefulness of the ICT in comparison with ELISAs for differential diagnoses in the laboratory. Because sera from patients with different pathologies, and not from healthy individuals, were used in this study, the diagnostic parameters provided by our analysis, including sensitivity and specificity, could not be generalized. Our focus was on the practicability of the test. The Em2-Em18-ELISA test provided the highest sensitivity, even if the specificity was not optimal. Routinely, the immunoblotting test is used to compensate for this deficiency. Detection of antibodies against the Em18 antigen has been demonstrated as a good marker for the study of *Echinococcus* activity in humans [13]. The rEm18-ICT and rEm18-ELISA have been proved useful in the laboratory to check therapy efficiency [19]. Based on serological follow-up and imaging (especially PET scan) [7], treatment can be adjusted on a case-by-case basis. Recent reports have indicated that, in some AE patients under long-term benzimidazole therapy, a parasitocidal effect may be observed [7]. If the activity of the parasite is indirectly checked by the immune response of the patient, it might be an indication that treatment should be ended.

The performance of the ELISA test combining Em2 and Em18 antigens was compared with rEm18-ELISA in a long patient surveillance study [2]. Both tests showed correlated results throughout the patient survey, with variations in immune activity paralleling curative or recurrence events after surgery.

For a manual test, because judgements are made based on visual observation, reproducibility is an absolute necessity, especially in the case of a weak immune response. Our findings on reproducibility indicated that slight differences in intensity can appear among ICT batches for the same serum sample presenting a weak immune response. When the rEm18-ICT is used in AE patient first screening tests or follow-up, two different operators must perform the reading and a reference sample should be included when the ICT is done. In our study the variability among the ELISA kit batches we used was also taken into account by including a reference sample in each ELISA series, and then calculating the index value based on this sample, as recommended by the manufacturer.

In conclusion, our findings demonstrate that the rEm18-ICT is a simple, reliable, and easy-to-use tool in AE diagnosis, requiring a minimum of equipment and time, especially for the first screening.
